# Atypical memory B cell clonal expansion and inflammatory programs associate with platelet-activating antibody development in COVID-19

**DOI:** 10.1172/jci.insight.201033

**Published:** 2026-02-26

**Authors:** Nathan Witman, Mei Yu, Yuqi Zhang, Kexin Gai, Yuhong Chen, Lu Zhou, Christine Nguyen, Wen Zhu, Yongwei Zheng, Shawn Jobe, Mary Beth Graham, Weiguo Cui, Demin Wang, Renren Wen

**Affiliations:** 1 Department of Microbiology & Immunology, Medical College of Wisconsin, Milwaukee, Wisconsin, USA.; 2 Versiti Blood Research Institute, Milwaukee, Wisconsin, USA.; 3 Department of Pathology, Northwestern University, Chicago, Illinois, USA.; 4 Department of Medicine, Medical College of Wisconsin, Milwaukee, Wisconsin, USA.

**Keywords:** Immunology, Vascular biology, B cells, COVID-19, Platelets

## Abstract

Patients with COVID-19 who develop platelet-activating antibodies represent a subset at heightened thrombotic risk, yet the immune features associated with this response remains to be defined. We applied single-cell RNA-seq of B and T cells, single B cell V(D)J-seq, and plasma cytokine and chemokine analysis to define immune signatures distinguishing patients who did (PEA^+^) or did not (PEA^–^) develop these antibodies. Patients positive for PEA showed prominent transcriptional enrichment of inflammatory, antigen presentation, and B cell receptor signaling pathways within antigen-experienced B cell subsets. Expanded B cell clones in patients positive for PEA were disproportionately enriched within atypical memory B cells and exhibited upregulated IFN-γ–response signatures, increased proliferative mutational patterns, limited class switching, and a significant overrepresentation of RKH/Y5 heavy-chain motifs associated with platelet-activating antibodies, consistent with an extrafollicular-biased response. Parallel T cell profiling revealed IL-12 pathway enrichment across most T cell subsets, increased IFN-γ transcription, and elevated plasma levels of Th1-associated cytokines in patients positive for PEA. Collectively, these data highlight a coordinated inflammatory environment marked by Th1-skewed T cell activation and selective expansion of atypical memory B cell clones carrying RKH/Y5 motifs, defining immunologic features associated with platelet-activating antibody development in COVID-19.

## Abstract 2

Immune profiling reveals inflammatory B- and T-cell signatures associated with platelet-activating antibodies in severe COVID-19, defining immune features linked to thrombotic risk.

## Introduction

Severe acute COVID-19 is often accompanied by serious thrombotic complications that lead to increased mortality (1). Dysregulated thrombosis in severe COVID-19 is commonly associated with platelet activation, thrombocytopenia, deep vein thrombosis, pulmonary embolism, and endothelial dysfunction (1, 2). Chronic coagulopathy has been reported in patients even after recovery from acute SARS-CoV-2 infection (3). Thrombotic complications in long COVID resemble those in acute infection, suggesting a shared mechanism (3). Notably, similar patterns of dysregulated thrombosis have also been documented in mRNA-based COVID-19 vaccines (4). These parallels suggest shared pathogenic pathways, underscoring the need to clarify the immune mechanisms driving thrombosis across infection, long COVID, and vaccination.

While thrombotic complications in COVID-19 are the result of a “perfect storm” involving multiple systems and factors (1, 2), we and others have investigated the potential contribution of antibodies (5–7). Severe patients with COVID-19 can develop complications resembling heparin-induced thrombocytopenia (HIT), a condition characterized by the formation of autoantibodies targeting platelet factor 4 (PF4)/heparin complexes, leading to platelet activation (8, 9). Previous work has shown that PF4-reactive antibodies can develop in patients with COVID-19 (5, 6). We have also shown that hospitalized patients with COVID-19 develop platelet-activating antibodies and that a subset of these antibodies bind to the receptor-binding domain of the SARS-CoV-2 spike protein (5). These findings suggest that B cell responses to SARS-CoV-2 antigens may drive platelet-activating antibodies and coagulopathy.

The origin and mechanisms behind the production of prothrombotic antibodies in COVID-19 remain unexplored. Patients with severe disease generate polyreactive antibodies that target both viral and host antigens (10, 11). These responses are accompanied by extrafollicular-biased B cell differentiation and expansion, and they are accompanied by enrichment of B cell populations with phenotypes that are variably described as atypical memory B cells, double-negative 2 B cells, or age-associated B cells, depending on context (10, 12, 13). These subsets have been implicated in autoimmune conditions, including systemic lupus erythematosus (SLE), and are thought to arise under inflammatory conditions, where they contribute to the generation of autoreactive antibody responses (14–16)

In severe COVID-19, the adaptive immune response is shaped by a dysregulated cytokine milieu. A key feature is the disruption of germinal center (GC) formation, attributed to the depletion of BCL-6–expressing T follicular helper cells (17). Concurrently, the inflammatory environment is marked by elevated levels of IFN-γ and widespread T cell activation (18–20). IFN-γ is a potent type 1 immunomodulatory cytokine that can drive certain extrafollicular (EF) B cell responses and has been associated with immune dysregulation in both infectious and autoimmune conditions (21, 22). Although these findings provide a framework for understanding the immunological disturbances in severe COVID-19, the specific pathways leading to the generation of prothrombotic, platelet-activating antibodies remain uninvestigated.

This study provides the first high-resolution, single-cell, and clonal characterization of immune features associated with platelet-activating antibodies in COVID-19. Using integrated scRNA-seq, V(D)J-seq, and cytokine profiling, we identified an inflammatory landscape in patients positive for P-selectin expression assay (PEA) (PEA^+^ patients) marked by enrichment of inflammatory gene sets across antigen-experienced B cell subsets. We also discovered prominent expansion within atypical memory B cell clonal families with increased IFN-γ signaling, mutational patterns consistent with heightened proliferation and activation, and enrichment for known platelet-activating motifs. Complementary analysis of T cells revealed increased IL-12 and paralleled inflammatory signaling pathways. Together, these findings highlight B and T cell immune states that accompany platelet-activating antibody development and establish framework for future mechanistic studies.

## Results

### B cells from patients with platelet-activating antibodies are enriched for inflammatory signaling pathways.

All patients from our cohort were admitted to the hospital upon confirmation of SARS-CoV-2 infection, and the presence of platelet-activating antibodies was determined using a modified PF4-dependent PEA (5). To best characterize what drives the development of platelet-activating antibodies, patients were selected based on having the highest and lowest PEA scores within our cohort of hospitalized patients (Supplemental Table 1; supplemental material available online with this article; https://doi.org/10.1172/jci.insight.201033DS1). Importantly, clinical and demographic characteristics were largely comparable between groups, with no differences in Sequential Organ Failure Assessment (SOFA) scores, a measure of disease severity, in PEA^+^ patients, despite significant differences in SpO_2_, PaO_2_, and C-reactive protein (Supplemental Table 1) (5). These data suggest platelet-activating antibody development is independent of disease severity but may be associated with inflammation.

**Figure 1:**
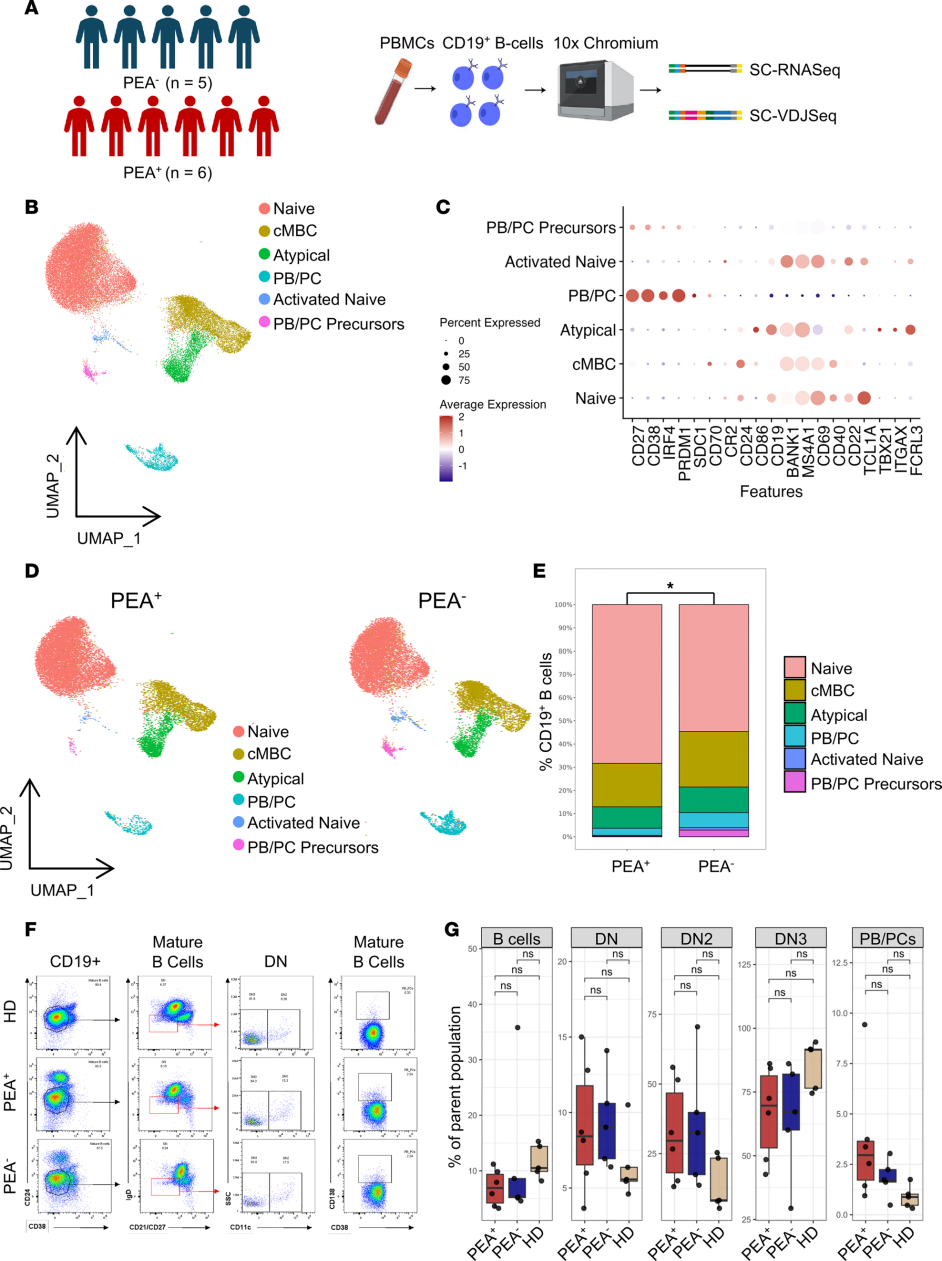
Comparable B cell clusters and subpopulations in PEA^+^ and PEA^–^ patients. CD19^+^ B cells were sorted by FACS from PBMCs of 6 PEA^+^ patients and 5 PEA^–^ patients, followed by scRNA-seq and scV(D)J-seq. (**A**) Schematic overview of patient selection and the scRNA-seq workflow. (**B**) UMAP visualization of CD19^+^ B cell clusters by gene expression. UMAP visualization of all CD19^+^ B cells (*n* = 29,505), colored by 6 identified clusters based on 5′ gene expression profiles and annotated according to B cell subset identities. (**C**) Dot plot showing the expression of key marker genes used to validate cluster annotations. (**D**) PEA status–specific UMAP of CD19^+^ B cells. UMAP plots from **B** split by PEA status, showing CD19^+^ B cells from PEA^+^ (*n* = 14,414) and PEA^–^ (*n* = 15,019) patients. (**E**) Comparison of B cell clusters between PEA^+^ and PEA^–^ patients represented as percentage of total CD19^+^ cells. Statistical analysis was performed using global multinomial logistic regression comparisons. (**F**) Representative flow cytometry gating strategy for B cell subsets between PEA^+^, PEA^–^, and healthy donor (HD) groups. Data represent a single experiment. (**G**) Flow cytometry analysis of B cell subsets between PEA^+^ (*n* = 6), PEA^–^ (*n* = 5), and HD (*n* = 5). Percents are presented of their parent populations gated from **F**. Statistical comparisons were generated with Wilcoxon rank-sum test with Bonferroni correction; *P < 0.05.

To better define the pathways driving prothrombotic, platelet-activating antibody production, we first performed paired single-cell RNA-seq (scRNA-seq) and 5′ V(D)J-seq (scV[D]J-seq) of peripheral blood B cells. We profiled CD19^+^ B cells from the peripheral blood of 6 PEA^+^ and 5 PEA^–^ patients using the 10x Genomics platform (Figure 1A and Supplemental Table 2). Preprocessing of scRNA-seq data and UMAP analysis yielded 13 Seurat clusters across all patients, and these clusters were subsequently consolidated into 6 final clusters by annotating them with established B cell population gene sets, including naive, activated naive, classic memory B cells (cMBC) and atypical memory B cells, plasmablast/plasma cell (PB/PC) precursors, and PB/PCs (Figure 1B and Supplemental Figure 1, A–E) (23–27). B cell subset annotations were validated by the expected expression of subset-specific transcripts and the corresponding immunoglobulin isotype (Figure 1C and Supplemental Figure 1, C and D). Although interpatient variability precluded significance at the individual-cluster level, multinomial logistic regression identified significant global differences in B cell distribution patterns, consistent with shifts observed by UMAP visualization (Figure 1, D and E, and Supplemental Figure 1F). Flow cytometry analysis yielded similar findings, with modest variation between the 2 COVID-19 patient cohorts that did not reach statistical significance (Figure 1, F and G, and Supplemental Figure 1G). Additionally, no changes were observed between healthy donors (HD) when compared with patients with COVID-19. Together, these findings indicate that the development of platelet-activating antibodies is not connected with broad alterations in B cell populations.

**Figure 2:**
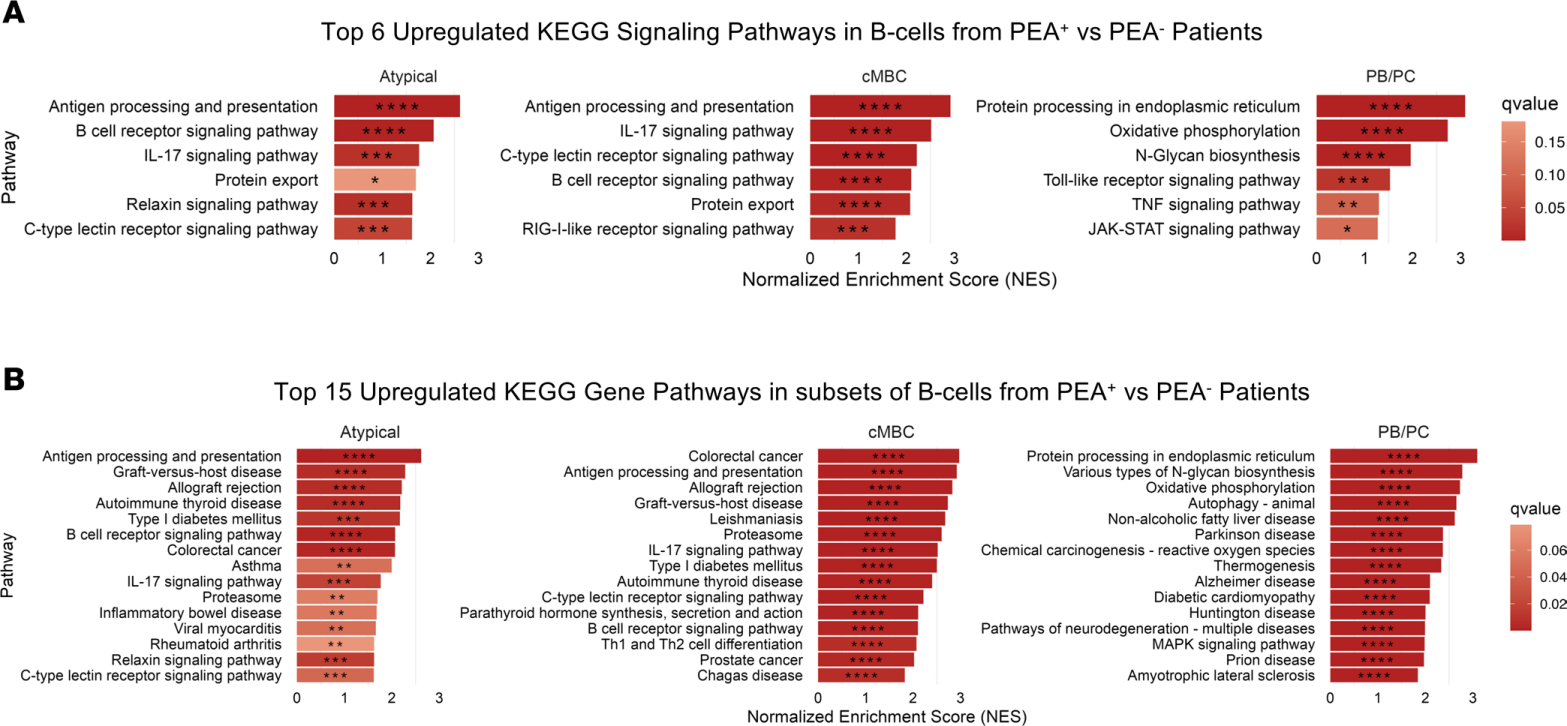
Elevated inflammatory pathway expression in PEA^+^ patients. GSEA was performed using a ranked gene list derived from pseudobulk-corrected differential expression analysis with DESeq2. Genes were ranked by log_2_ fold change, and enrichment was assessed against KEGG gene sets from MSigDB. (**A**) Top 6 KEGG signaling pathways enriched in B cell subsets of PEA^+^ patients compared with PEA^–^ patients. Plots display the top 6 signaling pathways enriched in atypical memory B cells, cMBCs, and PB/PCs from PEA^+^ patients, ranked by normalized enrichment score (NES). (**B**) Top 15 KEGG pathways enriched in B cell clusters of PEA^+^ patients relative to PEA^–^ patients. Plots display the top 15 enriched pathways in atypical memory B cell, cMBC, and PB/PC clusters of PEA^+^ patients, ranked by NES. Color intensity reflects FDR-adjusted *q* values, with statistical significance denoted as: *q < 0.25, **q < 0.1, ***q < 0.05, and ****q < 0.01.

To assess whether PEA positivity was associated with transcriptomic changes, we performed pathway-enrichment analysis of antigen-experienced B cell subsets using pseudobulk-corrected differential gene expression (DGE) values and KEGG gene pathway enrichment. This analysis revealed that, in PEA^+^ patients, atypical memory B cells and cMBCs showed enrichment of pathways related to B cell receptor, antigen-presentation, and cytokine- and innate immune–associated signaling (Figure 2A), along with KEGG pathways annotated as inflammatory- and autoimmune disease–associated pathways (Figure 2B). Moreover, PB/PC cells from PEA^+^ patients displayed enrichment of pathways related to protein synthesis, ER responses, cellular metabolism, and cytokine- and innate immune–associated signaling modules (Figure 2A), as well as disease-annotated KEGG pathways, including neurodegenerative and metabolic disorders (Figure 2B) (28–33). Individual DGE analysis identified a small number of significantly differentially expressed genes in PEA^+^ relative to PEA^–^ patients, including upregulation of *DIPK1B* in naive B cells and *SOCS3* in PB/PCs (Supplemental Figure 2) (34), as well as downregulation of *MPEG1* in cMBCs (Supplemental Figure 2).The limited magnitude and resolution of single-gene differences suggest that the development of platelet-activating antibodies is more likely to reflect subtle transcriptomic changes, rather than global reprogramming, of the responding B cell subsets.

### Expanded B cells in PEA^+^ patients exhibit upregulated IFN-γ signaling.

**Figure 3:**
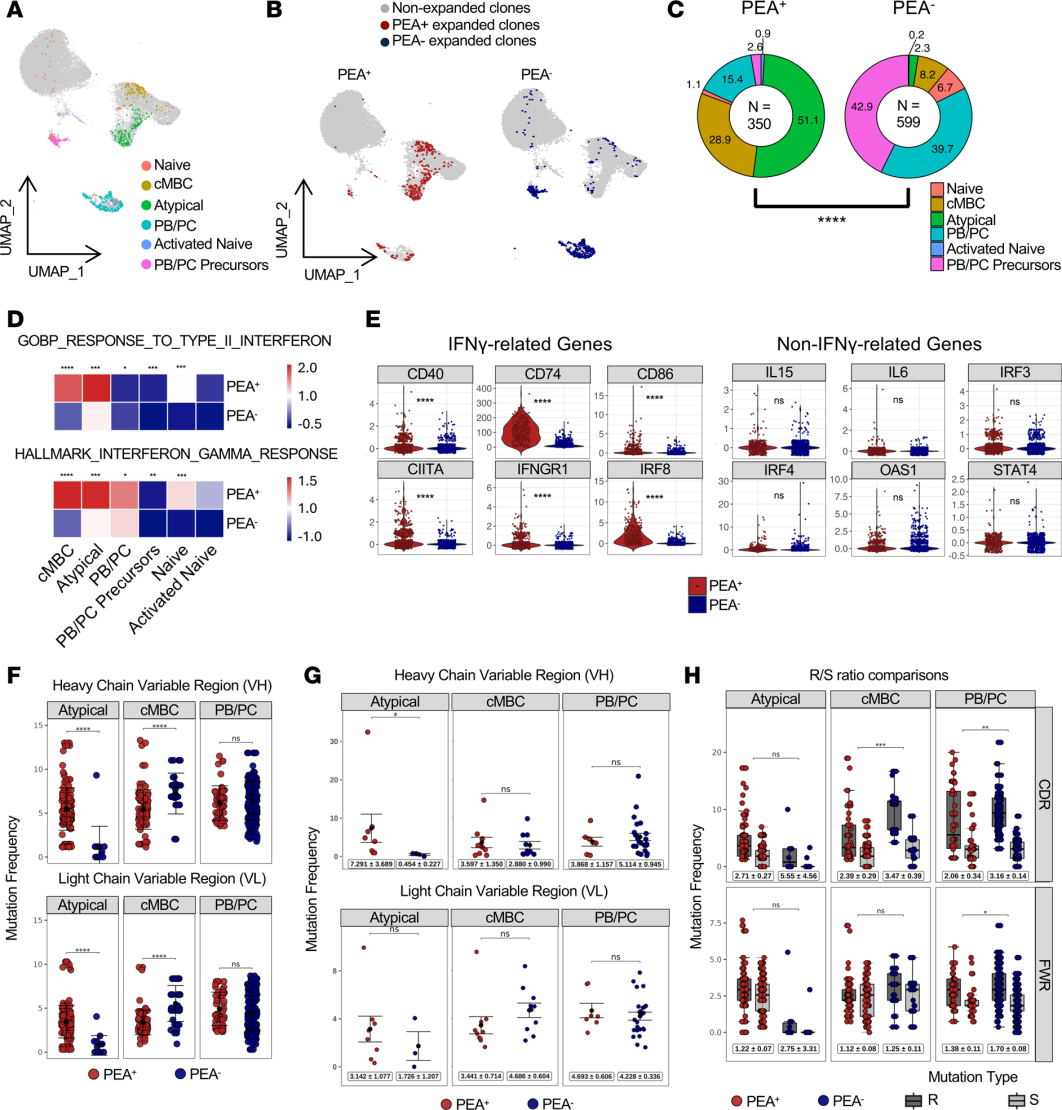
Evidence of IFN-γ upregulation and clonal atypical memory B cell expansion in PEA^+^ patients. (**A**) UMAP of 949 clonally expanded B cells, colored by cluster. Expanded clones were defined by shared heavy-chain CDR3 sequences with at least 7 cells per clone and were derived from 5 PEA^+^ and 3 PEA^–^ patients. (**B**) UMAP split by PEA status, highlighting expanded clones from PEA^+^ (*n* = 350) and PEA^–^ (*n* = 599) patients. (**C**) Distribution of expanded clones across B cell subsets. Donut charts show the relative percentage of expanded clones within each patient group, with slice size indicating abundance and color indicating cluster identity. Percentages denote each subset’s contribution to the total expanded pool. Statistical comparisons used a χ^2^ test. (**D**) IFN response signature scores for expanded clones across B cell subsets. Scores were calculated from GO:0034341 and HALLMARK_INTERFERON_GAMMA_RESPONSE gene sets, normalized as *z* scores, and displayed as a heatmap. Differences between PEA groups were assessed with the Wilcoxon rank-sum test. (**E**) Violin plots showing expression of IFN-γ–associated transcripts and selected control genes in expanded clones from PEA^+^ versus PEA^–^ patients. Statistics were computed using the Wilcoxon rank-sum test. (**F**) VH and VL mutation frequencies (total replacement [R] and silent [S]) in atypical memory B cells, cMBCs, and PBs/PCs, colored by PEA status. Data are shown as mean ± SEM. (**G**) Normalized total mutation frequencies for representative clones collapsed within each clonal family, plotted as average R+S mutations per clone, and colored by PEA status. Data are shown as mean ± SEM. (**H**) Replacement versus silent mutation frequencies and ratio comparisons in CDR and FWR regions across subsets. Data are shown as mean ± SEM. Statistical significance shown for PEA^+^ versus PEA^–^ comparisons; *P < 0.05, **P < 0.01, ***P < 0.001, ****P < 0.0001.

Because transcriptomic differences are observed in antigen-experienced B cells, and B cells producing platelet-activating antibodies in COVID-19 are diverse and hard to capture with known antigens (5), we focused on clonally expanded B cells as a marker of activation, an approach that offers a broader and more representative view of the activated B cell repertoire. B cells were grouped into clonal families if they originated from the same patient and shared the same V(D)J gene usage, identical CDR3 lengths, and at least 85% CDR3 nucleotide sequence identity. To minimize sequencing bias and random clonal occurrences, we defined expanded clones as clonal families comprising 7 or more cells (35, 36). Expanded clones were projected onto the original UMAP and stratified by PEA status for visualization (Figure 3, A and B). They exhibited distinct distributions between PEA^+^ and PEA^–^ patients: enrichment within activated naive, atypical memory B cells, and cMBC clusters in PEA^+^ individuals but mainly in PB/PC precursor and PB/PC clusters in PEA^–^ patients (Figure 3, B and C). Expansion of CD11c^+^ activated naive and atypical memory B cells has been linked to EF differentiation (37–39). Consistently, KEGG GSEA of expanded B cells revealed enrichment of gene signatures annotated to multiple disease-associated pathways, including autoimmune disease pathways (Supplemental Figure 3A), similar to patterns observed in the total B cell compartment (Figure 2, A and B). Many of these pathways are associated with EF B cell responses (40–42).

In multiple settings, EF B cell differentiation is regulated by IFN-γ signaling (37–39). To determine whether IFN-γ–associated transcriptional programs are enriched in expanded B cells in PEA^+^ patients, we assessed IFN-γ–responsive gene signatures. GSEA revealed significant enrichment of IFN-γ–response signatures across expanded B cell subsets, except for activated naive B cells, in PEA^+^ patients (Figure 3D) (43–46). Consistent with this enrichment, genes commonly included in IFN-γ–responsive transcriptional programs, including MHCII-associated molecules (*CIITA* and *CD74*) and other activation-associated genes (*CD86*, *IRF8*, and *IFNGR1*), were upregulated in expanded B cells from PEA^+^ patients (Figure 3E) (43–46). In contrast, we did not observe significant differences in the expression of several other immunomodulatory genes in PEA^+^ patients (Figure 3E). Although other inflammatory signaling pathways may also contribute, the overall expression pattern of these genes is consistent with a role for IFN-γ–associated signaling. Taken together, the combined pathway-level enrichment and gene-level expression patterns suggest a contribution of IFN-γ–associated signaling to B cell expansion in PEA^+^ patients.

### Somatic hypermutation and class-switch recombination features of expanded clones in PEA^+^ patients are suggestive of an EF origin of platelet-activating antibodies.

To better understand the development of these expanded B cells, we analyzed mutation frequencies within the variable regions of the heavy (VH) and light (VL) chains of antigen-experienced subsets. Expanded atypical memory B cells from PEA^+^ patients showed higher mutation frequencies, whereas cMBCs showed lower mutation frequencies relative to their counterparts in PEA^–^ patients (Figure 3F). Importantly, when expanded cells were normalized by collapsing clonal families into a single representative clone, this pattern persisted, most notably within the VH regions of atypical memory B cells (Figure 3G), indicating that these differences were not driven by clonal overrepresentation. The increased mutational burden in atypical memory B cells may be linked with the enriched B cell receptor signaling pathways (Figure 2A) and increased expansion of this subset (Figure 3C), consistent with a more activated and proliferative cellular state.

Analysis of selection pressure using the Immcantation framework within cMBC and atypical memory B cell subsets that showed mutation differences revealed a trend toward reduced selection pressure, particularly within the VH CDR3s in PEA^+^ patients (Supplemental Figure 3B). Consistent with this finding, analysis of replacement-to-silent mutation (R/S) ratios in expanded cells showed reduced R/S ratios across B cell subsets from PEA^+^ patients with statistically significant differences observed in cMBC and PB/PC populations (Figure 3H). Following clonal normalization, this pattern persisted but did not reach significance, likely reflecting limited clonal numbers (Supplemental Figure 3C). These findings suggest reduced selection pressure of expanded B cells in PEA^+^ patients compared with PEA^–^ patients.

Finally, analysis of isotype usage in expanded clones revealed a marked disparity; 79% of expanded clones in PEA^+^ patients were IgM^+^, compared with 19% in PEA^–^ patients (Table 1). In contrast, over 80% of expanded B cells in PEA^–^ patients have undergone class switching to IgA or IgG (Table 1), indicating substantially reduced class switching among expanded B cells in PEA^+^ patients. Collectively, the combination of increased proliferation, reduced class switching, and altered mutation burden in expanded atypical B cells from PEA^+^ patients is consistent with features previously associated with EF-biased B cell responses (22, 47).

### Expanded atypical memory B cells in PEA^+^ patients exhibit sequence signatures typical of platelet-activating antibodies.

**Figure 4:**
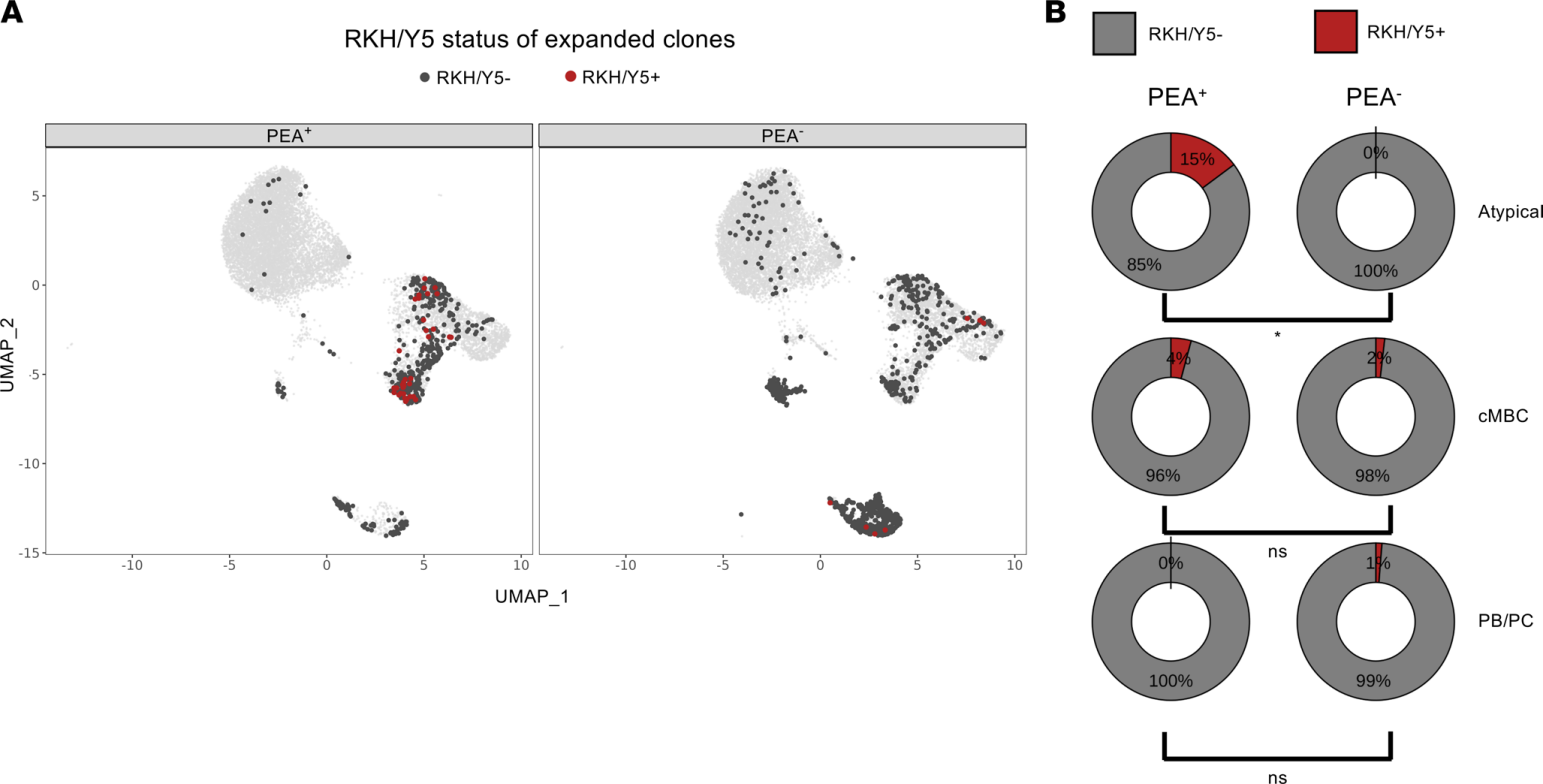
Expanded clones with sequence features characteristic of platelet-activating antibodies were specifically clustered within atypical memory B cells of PEA^+^ patients. (**A**) UMAP of expanded with ≥ 3 cells plotted on the original UMAP. Colors indicate the presence of RKH/Y5 motifs within each cell (dark gray, negative; red, positive) (*n* = 1,524). (**B**) Donut chart displaying the percentage of unique clone families with RKH/Y5 motifs within each cluster and split by PEA status (*n* = 238). Statistics were calculated using Fisher’s exact test; *P < 0.05.

In earlier studies, we identified structural motifs characteristic of platelet-activating antibodies, specifically R/K/H (RKH) or YYYYY (Y5) motifs within the HCDR3, in both COVID-19 and HIT (5, 48). We therefore explored whether B cells carrying these structural features preferentially expanded within atypical memory B cells in PEA^+^ patients. To ensure sufficient clones for comparison within clusters, we lowered the clonal family size threshold to 3 or more cells. We then projected expanded cells onto the parent UMAP, indicating whether the expanded cells had the RKH/Y5 motif (Figure 4A). Following clonal normalization, comparison of motif prevalence between PEA^+^ and PEA^–^ patients showed significant enrichment of clonal families possessing RKH/Y5 motifs within atypical memory B cells, but not other B cell subsets, in PEA^+^ patients (Figure 4B and Table 2). These data demonstrate that expanded B cells harboring platelet-activating motif are selectively enriched within atypical memory B cells in PEA^+^ patients.

### T cells show enrichment of inflammatory pathways in PEA^+^ patients.

**Figure 5:**
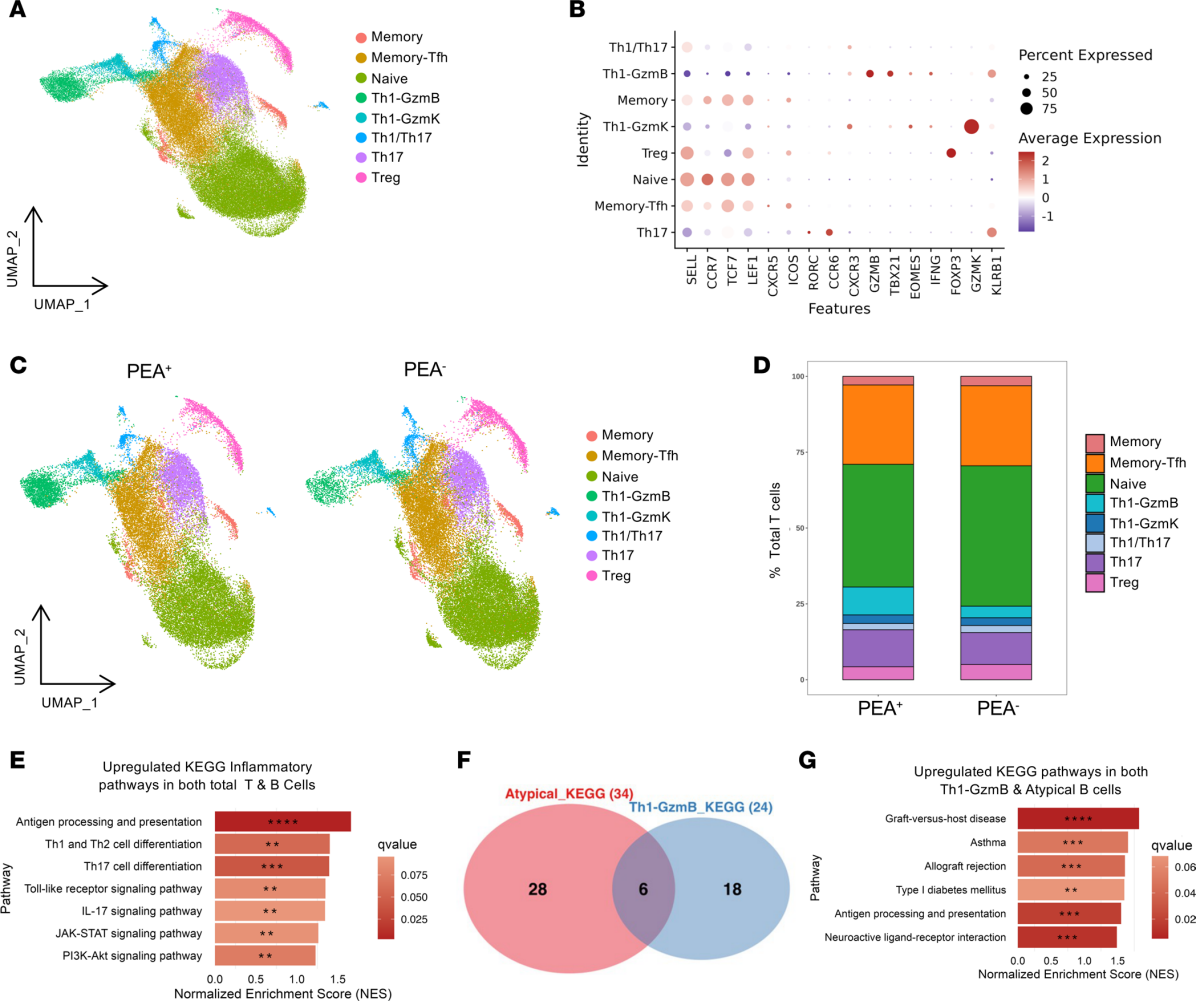
Paralleled upregulation of inflammatory programs in T cells from PEA^+^ patients. CD4^+^ T cells were FACS-sorted from PBMCs of 4 PEA^+^ and 5 PEA^–^ patients, followed by scRNA-seq. (**A**) UMAP visualization of CD4^+^ T cells. UMAP of 67,303 CD4^+^ T cells, colored by 8 clusters based on 5′ gene expression and annotated by subset identity. (**B**) Dot plot showing the expression of key marker genes used to validate the cluster annotations. (**C**) UMAP plots split by PEA status. UMAP plots show PEA^+^ (*n* = 30,930) and PEA^–^ (*n* = 36,373) cell distributions. (**D**) CD4^+^ T cell population distribution by PEA status, with PEA^+^ and PEA^–^. Significance was assessed by Wilcoxon rank-sum test. (**E**) GSEA on pseudobulk-adjusted DESeq2 gene rankings. Genes were ranked by log_2_ fold change, and enrichment was assessed using the MSigDB KEGG database. Comparative analysis of transcriptomes in CD4 T cells from PEA^+^ and PEA^–^ patients was performed for the indicated pathways. Color gradient indicates FDR-adjusted *q* values. *q < 0.25, **q < 0.1, ***q < 0.05, ****q < 0.01. (**F**) Overlapping KEGG pathways in atypical memory B cells and Th1-GzmB T cells from PEA^+^ patients. Venn diagram shows shared pathway enrichment between the 2 subsets types in PEA^+^ patients. (**G**) GSEA on pseudobulk-adjusted DESeq2 gene rankings. Genes were ranked by log_2_ fold change, and enrichment was assessed using the MSigDB KEGG database. Comparative analysis of transcriptomes in CD4 T cells from PEA^+^ and PEA^–^ patients was performed for the indicated pathways. Color gradient indicates FDR-adjusted *q* values. *q < 0.25, **q < 0.1, ***q < 0.05, ****q < 0.01.

To explore the cellular mechanisms driving atypical memory B cell expansion in PEA^+^ patients, we performed scRNA-seq of peripheral blood T cells from the same cohort of patients with COVID-19, including samples from 4 PEA^+^ and 5 PEA^−^ patients (Supplemental Table 3). Initial UMAP analysis of T cell transcriptomes from all 9 patients revealed 13 distinct clusters, which were subsequently consolidated into 8 major clusters (Figure 5, A and B, and Supplemental Figure 4, A–D). Subsequent stratification of the UMAP showed modest T cell population differences between PEA^+^ and PEA^–^ patients (Figure 5C), consistent with findings from the B cell analysis (Figure 1D). Importantly, PEA^+^ patients exhibited a trend toward higher frequencies of granzyme B^+^ Th1 cells (Th1-GzmB), although this did not reach significance, largely owing to variability driven by the COVID-79 sample (Figure 5D and Supplemental Figure 4E). Pseudobulk DGE analysis of T cells followed by pathway enrichment analysis identified 12 differentially expressed individual genes and revealed enrichment of KEGG-annotated inflammatory pathways in PEA^+^ patients, 7 of which were identical to those enriched in the total B cell GSEA (Figure 5E and Supplemental Figure 4F). Gene signature comparisons of pathway enrichment in atypical memory B cells and Th1-GzmB^+^ cells demonstrated overlapping upregulation of 6 autoimmune- and inflammation-associated pathways (Figure 5, F and G). These data suggest that both T and B cells in PEA^+^ patients may interact with and respond to similar inflammatory cues.

### T cells exhibit enrichment for IL-12 signaling pathways and IFN-γ gene expression in PEA^+^ patients.

**Figure 6:**
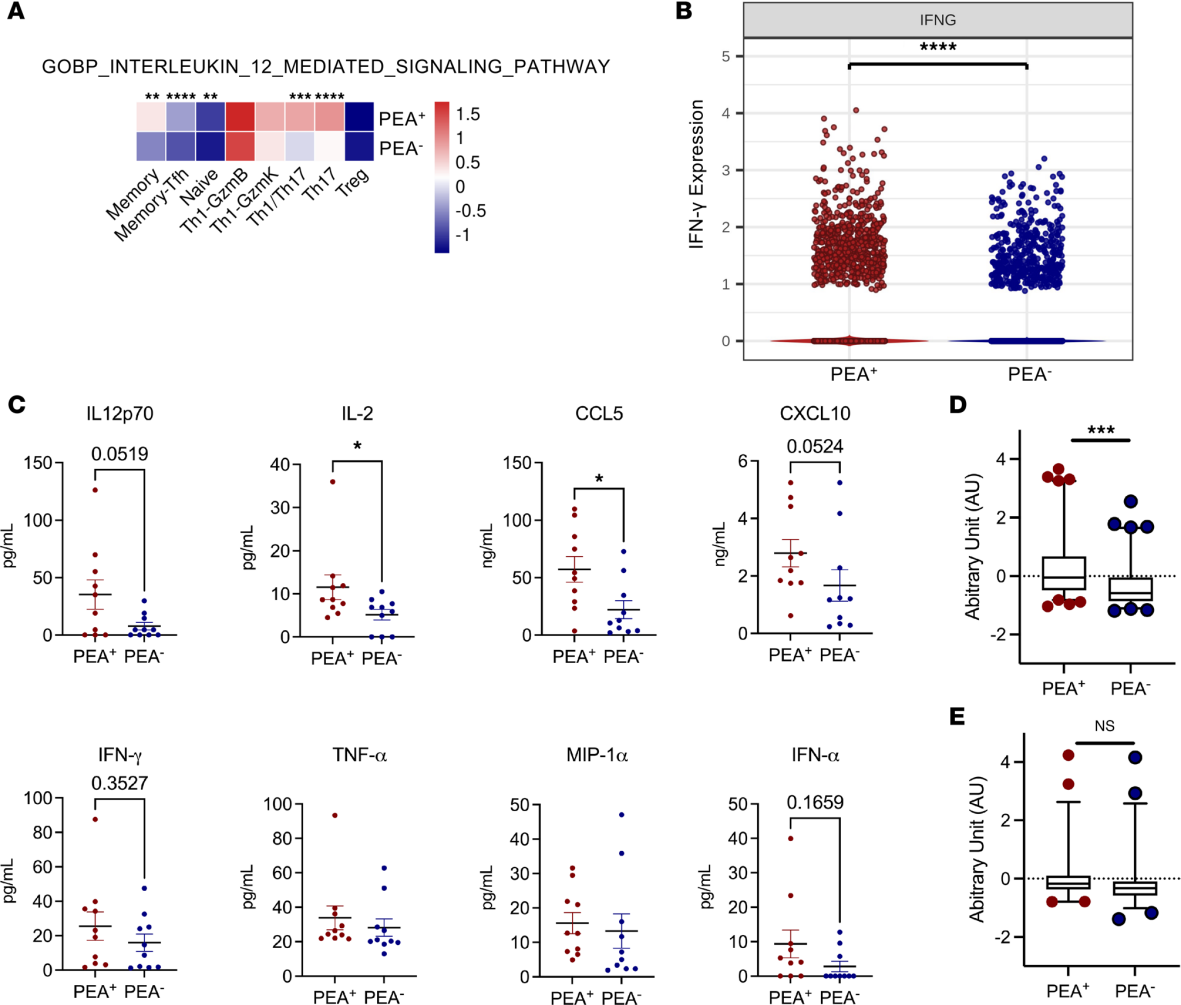
Increased IL-12 signaling pathway, IFN-γ, and Th1-associated cytokines in PEA^+^ patient T cells. (**A**) IL-12 signaling pathway enrichment scores using GOBP_INTERLEUKIN_12_MEDIATED_SIGNALING_PATHWAY (GO:0035722) normalized as *z* scores and displayed as a heatmap in T cell subsets. Significance was assessed by Wilcoxon rank-sum test. *P < 0.05; **P < 0.01; ***P < 0.001; ****P < 0.0001. (**B**) IFN-γ transcript levels compared between total T cells from PEA^+^ and PEA^–^. Significance was determined by Wilcoxon rank-sum test; ****P < 0.0001. (**C**) Plasma cytokine comparison between PEA^+^ and PEA^–^ patients. Plasma cytokine levels were compared between PEA^+^ (*n* = 10) and PEA^–^ (*n* = 10) patients. Statistical comparisons were conducted using Mann-Whitney *U* tests; *P < 0.05. (**D**) Cumulative analysis after standardization of the 8 inflammatory cytokines shown in **C** in the plasma of 10 PEA^+^ and 10 PEA^–^ patients with COVID-19. Box plot showing the scaled and centered results of each cytokine fit with a linear model and adjusted with estimated marginal means. (**E**) Cumulative analysis after standardization of the 5 inflammatory cytokines not related to Th1 responses in the plasma of 10 PEA^+^ and 10 PEA^–^ patients with COVID-19. Box plot showing the scaled and centered results of each cytokine fit with a linear model and adjusted with estimated marginal means. Statistical comparison was conducted using type III ANOVA F-test in the emmeans R package; ***P < 0.001.

Because Th1 T cells can drive atypical memory B cell development and EF responses, we examined whether PEA^+^ patients show transcriptomic evidence of Th1 skewing by first assessing enrichment of IL-12 signaling pathways. All but 3 T cell subsets in PEA^+^ patients showed significant enrichment of IL-12 signaling (Figure 6A), suggesting an IL-12–rich milieu that may promote Th1-GzmB^+^ T cell differentiation in these patients, consistent with the established essential role of IL-12 in Th1 cell differentiation (22, 49). We next assessed whether IFN-γ transcripts were elevated in PEA^+^ T cells. At the population level, total T cells from PEA^+^ patients exhibited increased IFN-γ expression (Figure 6B). To identify the subsets contributing most prominently to this signal, we quantified IFN-γ transcript levels across T cell subsets and found that Th1-GzmB^+^ cells were the highest expressors (Supplemental Figure 5A). When comparing PEA^+^ versus PEA^–^ patients within each subset, IFN-γ expression was significantly increased in naive T cells, with directional increases observed across additional subsets, consistent with the enrichment of IL-12 signaling pathways in total PEA^+^ T cells (Supplemental Figure 5B). Taken together, these data demonstrate that PEA^+^ patients show enrichment of IL-12 signaling across multiple T cell subsets and increased IFN-γ expression in total T cells, supporting the presence of a more pronounced Th1-skewed transcriptional profile in these patients.

To evaluate whether the plasma protein profile reflected a Th1 response, we quantified cytokines and chemokines that either induce or are produced by Th1 cells during inflammatory responses (IL-12, IL-2, CCL5, CXCL10, IFN-γ, IFN-α, MIP-1α, TNF-α) (50–54) using multiplex assays (Figure 6C). Although all the tested cytokines trended toward an increase in PEA^+^ patients, IL-2 and CCL5 were the only ones that reached significance, with IL-12 and CXCL10 nearing significance at *P* = 0.0519 and 0.0524, respectively (Figure 6C). Given the heterogeneity in patients and our small sample size, we generated a standardized composite score of these analytes, which showed a significant increase in plasma inflammatory response related to T cell activation in PEA^+^ patients (*P* < 0.001) (Figure 6D). By contrast, composite scores of inflammatory mediators unrelated to T cells (APRIL, BAFF, IL-6, IL-7, and CXCL13) did not differ between groups (Figure 6E). Together, these findings indicate upregulation of inflammatory and Th1-associated plasma mediators in PEA^+^ patients, supporting an enhanced inflammatory response that may contribute to Th1-skewed transcriptomic enrichment.

## Discussion

In this study, we performed single-cell transcriptomic analysis of B and T cells, paired B cell scV(D)J-seq, flow cytometry, and plasma cytokine/chemokine profiling to investigate the origin of prothrombotic antibodies in COVID-19. Integrated analyses demonstrated enrichment of inflammatory pathways in PEA^+^ patients. These patients showed selective expansion of atypical memory B cell clones with impaired class switching, increased mutation frequencies, elevated IFN-γ signaling, and enrichment of known platelet-activating antibody–associated RKH/Y5 motifs specifically within atypical memory B cells. Consistently, Th1-GzmB^+^ T cells were increased in the majority of PEA^+^ patients, and multiple T cell subsets exhibited enhanced inflammatory and IL-12 signaling pathways. PEA^+^ patients also displayed significant enrichment of composite scores of inflammatory and Th1-associated plasma cytokines/chemokines, supporting an enhanced inflammatory Th1-skewed response. This inflammatory milieu may contribute to the clonal expansion of atypical memory B cells, consistent with prior studies showing that IL-12 and IFN-γ can promote EF responses (21).

Robust EF B cell responses and impaired GC formation are associated with severe COVID-19 and increased mortality (10, 17). In addition, widespread T cell activation and elevated IFN-γ levels in severe COVID-19 have been shown to promote atypical memory B cell and plasmablast expansion (18, 19). EF responses in COVID-19 have also been shown to give rise to autoantibodies commonly observed in autoimmune diseases, including SLE, rheumatoid arthritis, and systemic sclerosis (11). However, whether and how these EF-driven autoantibodies, typically implicated in chronic tissue damage in autoimmune diseases, contribute to acute organ injury and mortality in COVID-19 are not completely understood. Our study suggests that, in a subset of patients, inflammation, Th1-skewed T cell responses, and atypical memory B cell expansion are associated with prothrombotic antibody responses and may underlie severe COVID-19. These data support a model linking EF B cell and Th1 T cell responses in COVID-19–related morbidity and mortality.

Atypical memory B cells from PEA^+^ patients exhibited elevated mutation levels, similar to those observed in cMBCs (Figure 3F). This unique mutation pattern has also been reported in *Plasmodium* and *Salmonella* infections, both of which are associated with strong EF responses (13, 55–57). These infections, as well as HIV and SLE, are also characterized by autoantibodies and EF responses (13, 58–61). Additionally, the elevated mutation frequency in atypical memory B cells occurred in the context of enriched B cell receptor signaling pathways, marked cell expansion, and impaired class switching, all of which are consistent with proliferation-associated mutation characteristic of EF B cell responses. Furthermore, IL-12 and IFN-γ are central in promoting an EF response (21). Importantly, many infectious and autoimmune conditions, including HIV, malaria, and SLE, are frequently associated with coagulopathy akin to that in severe COVID-19, such as thrombocytopenia and thrombosis (62–64).Together, these parallels further support a model by which inflammation, Th1 responses, and EF B cell responses lead to the development of prothrombotic platelet-activating antibodies and raise the possibility that platelet-activating antibodies may be more broadly present across infectious and autoimmune settings.

This study has several limitations. Foremost, this was a small cohort study with 11 patients, limiting statistical power to detect subtle population-level differences and increasing susceptibility to interpatient variability, including the influence of outliers and limiting generalizability. While EF and GC dynamics primarily occur in secondary lymphoid tissues, our conclusions rely on transcriptional and clonal signatures derived from peripheral blood rather than direct tissue-based analysis. Moreover, the observational nature of the study precludes mechanistic inference; although we identified associations between Th1-related inflammation, atypical memory B cell expansion, and RKH/Y5 motif enrichment, we cannot infer causality. Finally, the motif-based approach identifies structural features associated with platelet-activating antibodies but does not directly confirm antigen specificity or functionality.

Despite these limitations, the integrated single-cell and cytokine analyses reveal a consistent immunologic pattern in PEA^+^ patients: Th1-skewed inflammation with enrichment of IL-12 pathways and IFN-γ expression, as well as prominent expansion of atypical memory B cell clones enriched for platelet-activating antibody–associated motifs and increased mutation burden. These findings support a model in which inflammatory cues shape the B cell response, favoring EF responses and the emergence of platelet-activating antibodies. This B cell activation may not be dependent on large-scale population changes but rather on the composition of the B cell repertoire. Future work incorporating lymphoid tissue analyses, longitudinal sampling, and antigen-specific functional studies will be essential to fully define the origin and mechanisms by which prothrombotic antibodies develop in COVID-19.

## Methods

Supplemental Methods are available online with this article.

### Sex as a biological variable.

Both male and female patients were utilized in this study. Samples were not chosen based on sex, as sex was not considered as a biological variable in this study.

### Sample collection.

All patients were confirmed COVID-19^+^ via reverse transcription PCR testing. Blood was collected from patients prior to plasma administration on day 0. Patients were defined as either PEA^+^ or PEA^–^ based on Zhu et al. (5) Six PEA^+^ patients and 5 PEA^–^ patients were selected for scRNA-seq. Patient information can be found in Supplemental Tables 1–3.

### Flow cytometry.

Cryopreserved PBMCs were thawed, washed, and resuspended in FACS buffer (PBS with 2% FBS). A total of 1 × 10^6^ PBMCs per sample was centrifuged at 350 x g and resuspended in 75 μL FACS buffer together with 5 μL Fc receptor–blocking reagent (BioLegend, 422302) for 5 minutes at room temperature. For staining anti-IgG, it was reported Fc block interfered with staining (10); therefore, these samples were first incubated with the anti-IgG antibody alone for 5 minutes at 22°C prior to addition of the blocking reagent. After blocking, 25 μL of the antibody cocktail (Supplemental Table 4) was added to each sample (final staining volume, 100 μL), followed by incubation for 20 minutes at 4°C. Cells were washed with PBS and stained with Zombie NIR fixable viability dye (BioLegend, 423106) diluted in PBS. After a second wash, cells were fixed in 0.8% paraformaldehyde for 10 minutes at 22°C in the dark, washed, and resuspended for analysis. Samples were acquired on a Cytek Aurora full-spectrum cytometer using Cytek SpectroFlo software. One million events per sample were collected. Data were analyzed using FlowJo v11 (FlowJo LLC).

### Pseudobulk-corrected DGE and KEGG pathway analysis.

To remove inherent skewing within patient samples, pseudobulk-corrected gene expression analysis was conducted. RNA counts were extracted and converted into a single-cell experiment (65). Aggregated expression values from each cluster within each patient were summed up using “AggregateExpression” function, and PEA^+^ versus PEA^–^ patient values were compared on the sample level using “DESeq2” (66). Significance was determined with adjusted *P* ≤ 0.05. Preranked gene lists with fold change levels high to low were used for gene set enrichment analyses (GSEA) using clusterProfiler (v4.12.6) (67). The KEGG database was utilized in this analysis. To control the FDRs in both differential expression and GSEA, the Benjamini-Hochberg method was employed to adjust *P* values (68).

### HALLMARK and GO pathway analysis.

IFN-γ and IL-12 pathways were first identified, and their genes were isolated using both Hallmark and GO databases utilizing “msigdbr” (69, 70). Module scoring was then applied to each cell based on their expression of genes within each gene set by utilizing “AddModuleScore”. The average of each cluster and patient cohort was then calculated and normalized by generating scaled gene set scores. Differences were then plotted utilizing “pheatmap”.

### B cell receptor V(D)J data processing.

Demultiplexed FASTQ files from scV(D)J libraries were aligned to human V(D)J reference using the “cellranger vdj” command in cellranger (v7.0.1). For cells with multiple IgH or IgL hits, only 1 IgH and 1 IgL with the highest UMI were kept for the analysis. V(D)J data were then integrated into the scRNA-seq Seurat object as metadata for further analysis. The 10X V(D)J data were then processed using the Change-O toolkit to assign clones (71). Briefly, 10X V(D)J data were first converted to AIRR rearrangement data using “AssignGenes.py” and “MakeDb.py”. After splitting the IgH and IgK/L chain data, clonal groups were assigned based on heavy chain information and then corrected using light chain data. Structural motifs were determined as previously described (5, 72), but briefly, we determined RKH/Y5 possessing clones as those that have 3 or more cationic residues (R/K/H) or YYYYY motifs present within the heavy chain CDR3.

### B cell clonal analysis.

Combined V(D)J files were analyzed using the Change-O package from the Immcantation group (https://immcantation.readthedocs.io/en/stable/) to assign V(D)J regions, draw V(D)J boundaries, reconstruct germline sequences, and assign cells to clonal clusters (71). Clones were determined by utilizing 85% sequence similarity within the heavy-chain CDR3 region. Clone comparisons were determined by utilizing the Alakazam package from the Immcantation group (71). To determine the number of cells within each clonal group, the function “countClones” was utilized. For cluster analysis, maximally expanded clones were selected based on having > 6 cells with the same clone ID, and clones for expression comparison were selected based on having > 2 cells (Supplemental Table 2).

### Somatic hypermutation and selection pressure analysis.

Mutation frequencies for IgH and IgL chain and the CDR1-3 and FWR1-4 regions were determined utilizing the SHazaM package from the Immcantation group (71, 73). The selection pressure was then calculated using the “calcBaseline” and “groupBaseline” functions. Statistical comparisons were made using “testBaseline”, and plots were generated using “plotBaselineSummary”.

### Cytokine multiplex analysis.

Plasma proteins were measured using a multiplex Luminex assay as previously described (5). Briefly, EDTA plasma was diluted 1:2 and assayed in duplicate using the Premixed Multi-Analyte Kit Luminex Discovery Assay (Bio-Techne; LXSAHM). Plates were read on a Luminex FlexMap 3D analyzer.

### Statistics.

Statistical analyses were performed using R (version 4.4.1). A 2-sided *P* value less than 0.05 was considered statistically significant unless otherwise indicated. For comparisons between 2 independent groups, nonparametric Wilcoxon rank-sum tests were used, while paired comparisons were assessed using Wilcoxon signed-rank tests. Comparisons involving categorical variables were evaluated using Fisher’s exact test. For analyses involving more than 2 groups, *P* values were adjusted for multiple comparisons using the Benjamini-Hochberg FDR correction. scRNA-seq DGE analyses were performed on pseudobulked data to account for within-patient correlation, and pathway enrichment analyses were evaluated using GSEA with FDR correction. Multinomial logistic regression was used to assess differences in cell-type distributions across conditions while accounting for interpatient variability. No samples were excluded from analysis unless they failed predefined quality-control criteria for sequencing depth, cell viability, or antibody staining, as detailed in the Methods. Outliers were not excluded unless explicitly stated. Sample sizes and statistical tests used are indicated in the figure legends.

### Study approval.

Eleven patients with COVID-19 hospitalized at Froedtert Hospital were entered in the open-label clinical trial Evaluating the Efficacy and Safety of High-Titer Anti-SARS-CoV2 plasma in hospitalized patients with COVID-19 infection (NCT04354831). The study was approved by the IRBs of the Medical College of Wisconsin and Froedtert Hospital.

### Data availability.

All data supporting the findings of this study are available within the article and its Supporting Data Values file. The scRNA-seq and paired V(D)J-seq datasets generated in this study have been deposited in the NCBI BioProject database under accession number PRJNA1391649. Supporting analytic code used for data processing and analysis is available from the corresponding author upon reasonable request.

## Author contributions

SJ, MBG, WC, DW, and RW established the COVID-19 patient study group. NW and MY performed B and T cell bioinformatic analyses and made the final figures. Y Zhang and KG performed the initial T cell bioinformatic analysis. MY, YC, LZ, and CN carried out scRNA-seq and V(D)J-seq experiments. NW and WZ performed plasma protein and antibody analyses, respectively. MY, CN, WZ, and Y Zheng contributed to sample fractionation and storage. RW conceived the project and directed the studies. NW drafted the manuscript. NW, RW, DW, and MY made substantial revisions. All authors contributed to and approved the final version.

## Conflict of interest

The authors have declared that no conflict of interest exists.

## Funding support

This work is the result of NIH funding, in whole or in part, and is subject to the NIH Public Access Policy. Through acceptance of this federal funding, the NIH has been granted the right to make the work publicly available in PubMed Central.

- NIH grant HL148120 (RW)
- NIH grant HL161127 (RW)
- NIH grant HL167668 (RW and DW)
- NIH grant AI079087 (DW)
- NIH grant HL130724 (DW)
- Versiti Blood Center Research Foundation (RW and DW)
- American Heart Association (AHA) predoctoral fellowships 25PRE1374068 (LZ) and 20PRE35210461 (WZ)

## Supplementary Material

Supplemental data

Supporting data values

## Figures and Tables

**Table 1:**
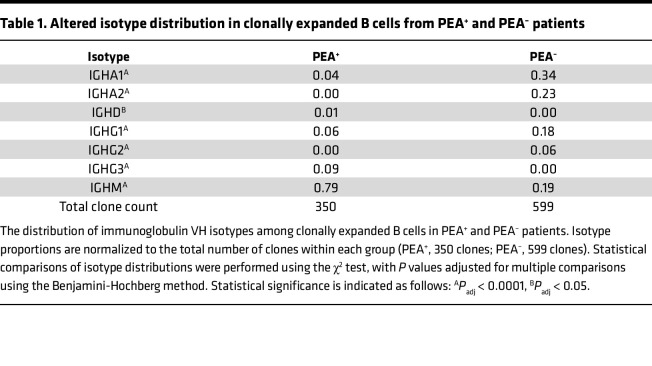
Altered isotype distribution in clonally expanded B cells from PEA^+^ and PEA^–^ patients

**Table 2:**
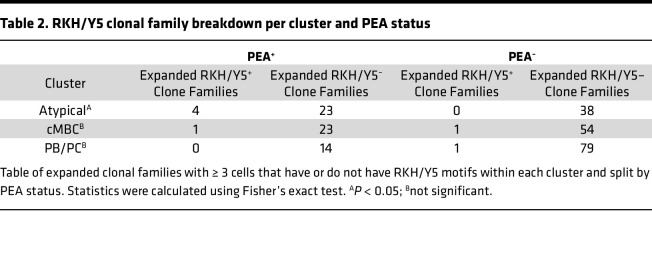
RKH/Y5 clonal family breakdown per cluster and PEA status

## Version 1 02/26/2026

In-Press Preview

## Version 2 04/22/2026

Electronic publication
