# Supplementary material for: Atypical memory B cell clonal expansion and inflammatory programs associate with platelet-activating antibody development in COVID-19
Source: JCI Insight. 2026 Feb 26;11(8):e201033. doi: 10.1172/jci.insight.201033 (PMC13135393; doi:10.1172/jci.insight.201033)
Supplement: Supplemental data [file jciinsight-11-201033-s054.pdf]

Supplemental Materials

Atypical memory B-cell clonal expansion and inflammatory programs associate with platelet-activating antibody development in COVID-19.

Nathan Witman[1,2]*, Mei Yu[2]*, Yuqi Zhang[3], Kexin Gai[3], Yuhong Chen[2], Lu Zhou[1,2], Christine Nguyen[1,2],

Wen Zhu[1,2], Yongwei Zheng[2], Shawn Jobe[2], Mary Beth Graham[4], Weiguo Cui[1,2†], Demin Wang[1,2], Renren Wen[1,2§]

[1]Department of Microbiology & Immunology, Medical College of Wisconsin, Milwaukee, WI, USA.

[2]Versiti Blood Research Institute, Milwaukee, WI, USA.

[3]Department of Pathology, Northwestern University, Chicago, IL, USA.

[4]Department of Medicine, Medical College of Wisconsin, Milwaukee, WI, USA

[†]: Current address: Department of Pathology, Northwestern University, Chicago, IL, USA.

*: These authors contributed equally to this study

[§]: Corresponding author, rwen@versiti.org

Supplemental Methods

PBMC Isolation and cryopreservation: Whole blood from COVID-19 patients was collected with EDTA tubes and spun down at 1000 rpm for 10 min to remove the plasma. The remaining EDTA blood was carefully layered onto Lymphoprep (Stemcell) at a 2:1 ratio in a 15ml tube and centrifuged at 800 g for 30 min without braking. Buffy coats were washed once and resuspended in 2X resuspension media (40% FBS in RPMI) before being transferred into cryovials. 2X freezing media (40% FBS + 30% RPM + 30% DMSO) were added to EDTA-PMBCs at a 1:1 ratio in cryovials, which were stored in Mr. Frosty filled with isopropyl alcohol and directly transferred to a –80 °C freezer. Cryovials stored at –80 °C freezer was then transferred into a nitrogen tank for long-term storage.

Sample preparation Frozen PMBCs were recovered following the instructions of 10X Genomics (https://cdn.10xgenomics.com/image/upload/v1710525012/CG000447_Handbook_CellThawingProtocols_SingleCellAssays_Rev_B.pdf). Briefly, cryovials were incubated at 37°C immediately until little ice remained. Cells were transferred into a 50 ml conical tube and vials were rinsed with 1mL warm medium, which was then transferred into a 50 ml conical tube using wide-bore pipette tips drop-wise. Cells were sequentially diluted by adding warm medium at the speed of 1 ml/3-5 sec to the tube and swirl for a total of 5 times. Cells were then centrifuged at 300 rcf for 5 min. Supernatant was removed and cells were resuspended in 1ml remaining media. 9ml warm media was added at the speed of 1 ml/3-5 sec into the tube, which was then spun at 300 rcf for 5min. After removal of the supernatant, cells were then resuspended in 1 ml PBS + 0.04% BSA. Cell concentration was determined, and cells were resuspended at desired concentrations. PBMCs were then stained with human anti-CD19 and anti-CD4 antibodies. $CD19^+$ B-cells and $CD4^+$ T-cells were sorted by FACS. Purified B-cells and T-cells were washed and resuspended in PBS + 0.04% BSA.

10X cDNA libraries preparation and sequencing: cDNA and V(D)J libraries for scRNA-seq analysis were prepared following the instructions of 10X Genomics

(https://cdn.10xgenomics.com/image/upload/ v1722286086/support-documents/CG000330_Chromium_Next_ GEM_Single_Cell_5_v2_Cell_Surface_ Protein_UserGuide_RevG.pdf). Briefly, cells diluted at the desired concentration were mixed with reaction mix on Chromium GEM Chip K to generate GEM and barcode the cells. After GEM-RT, GEMs were broken, and 10X barcoded first-strand cDNA was purified for each sample. Full-length cDNA was then amplified and purified to further generate V(D)J and 5' Gene Expression libraries, following the instructions, respectively.

Pre-processing of sequencing data: Demultiplexed FASTQ files from Gene Expression libraries were firstly aligned to GRCh38 human reference genome to generate matrix files. Matrix files were loaded into Seurat (V5.2.1) in R (V4.4.2) to generate Seurat objects for each sample. Data of each sample were preprocessed with a filter (200< feature number < 3500 & pt.mt < 10%) to remove doublet or dead cells. IgHV, IgHD, IgHJ, IgLV, IgLJ-related genes were regressed out before SCTransform was performed. *VariableFeatures* of each sample was set to 3000. After "*SelectIntegrationFeature*" and "*FindIntegrationAnchors*", data from individual patients were integrated together to remove batch effects among samples. Data from all samples were scaled to regress out cell cycle-related features. PCA and UMAP were run on the integrated dataset, and 16 clusters were identified eventually. RNA values were normalized using "*LogNormalize*" from the Seurat package. Variable features were then identified using Seurat's "*FindVariableFeatures*" with the number genes set to 3000 and using the statistical method "vst". Samples were integrated using "*SelectIntegrationFeatures*" by using 3000 genes and utilizing "*FindIntegrationAnchors*" with the dimensions set to 1:50 and finally combining the samples into a single Seurat object using "*IntegrateData*". Principal component analysis was then conducted using 50 principal components, followed by the "*RunPCA*" function. This was then supplemented by generating a UMAP using the "*RunUMAP*" function, with dimensions set to 1:50. Clusters were subsequently determined using the "*FindNeighbors*" and "*FindClusters*" functions. The "*SingleR*" (V2.6.0) package was used to annotate

and remove contaminating cells, and ultimately resulted in 13 B-cell clusters[1]. Previously published gene sets were then generated and compared to outputs from "*FindMarkers*" to properly annotate each B-cell cluster.

Supplemental Figures

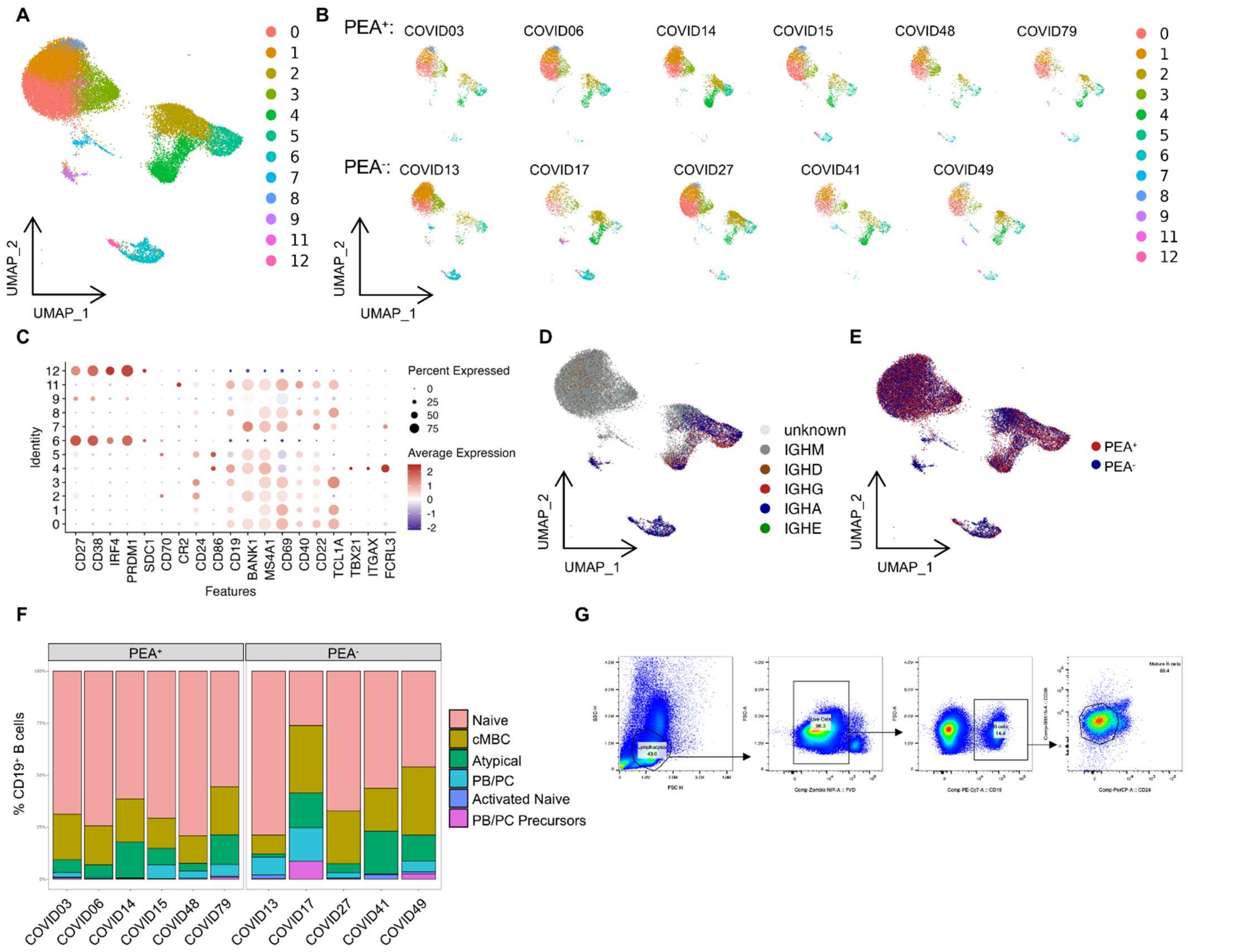

Supplemental Figure 1. B-cell UMAP verification and validation. (A) UMAP showing original B-cell clusters 0–12 from both PEA⁺ and PEA⁻ patients (n = 29,505). **(B)** UMAP split by Patient ID. **(C)** Marker expression used for cluster verification shown across the 13 original clusters. **(D)** Isotype distribution projected onto the UMAP. **(E)** Integration validation by grouping cells according to PEA status.**(F)** Distribution of B-cell populations percentages per patient, grouped by PEA status.**(G)** Gating strategy for identifying mature B-cells. Fixed viability dye = FVD.

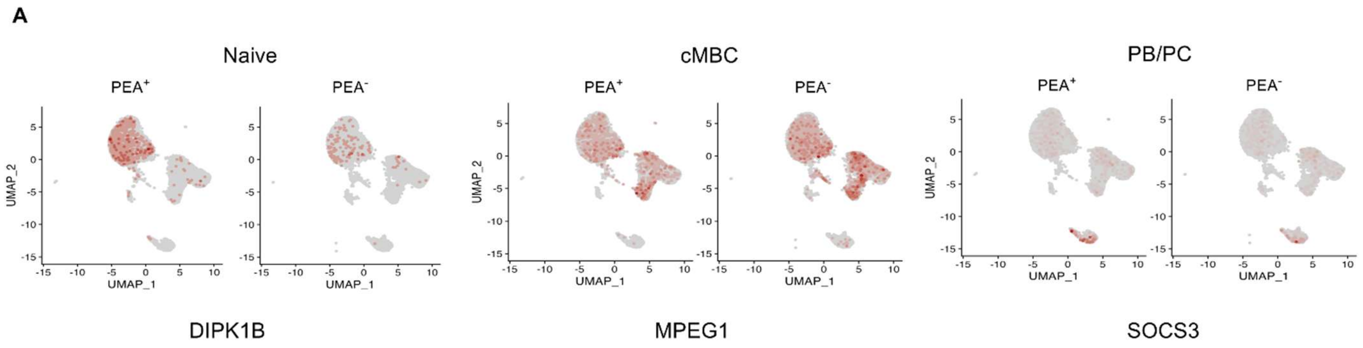

Supplemental Figure 2. Comparative GSEA of pseudobulk gene expression between PEA+ and PEA− patients. (A) Significantly upregulated genes, DIPK1B, MPEG1, and SOCS3, were identified in the naïve B-cell, cMBC, and PB/PC clusters, respectively. Significance was defined as having an adjusted p-value of 0.05 or less from DESeq2 pseudobulk-corrected differential expression analysis.

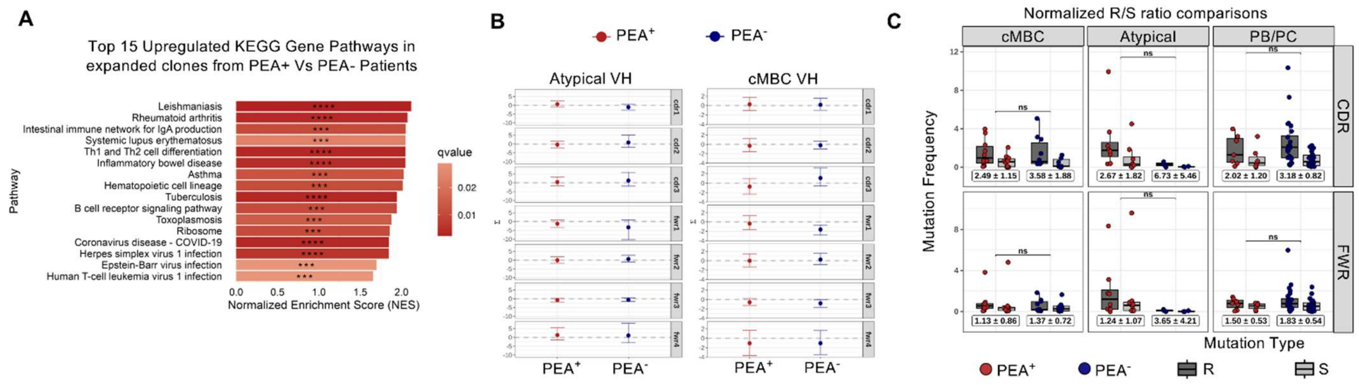

Supplemental Figure 3. Inflammatory pathway enrichment and somatic mutation features of expanded B-cell clones in PEA$^+$ patients. Expanded clones were defined as those with seven or more cells and identified in 5 PEA$^+$ and 3 PEA$^-$ patients. **(A)** Enrichment was assessed using preranked GSEA against the KEGG database. The top 15 positively enriched pathways in PEA$^+$ expanded cells are shown, ranked by NES. Statistical significance was determined using FDR-adjusted q-values; *q < 0.05, **q < 0.01, ***q < 0.001, ****q < 0.0001. **(B)** Selection pressure analysis to evaluate replacement-to-silent (R/S) mutation patterns in immunoglobulin heavy-chain variable regions. Analysis was performed separately for complementarity-determining regions (CDRs) and framework regions (FWRs) within expanded B-cell clones across classic memory B-cells (cMBCs), Atypical memory B-cells, and plasmablast/plasma cell (PB/PC) subsets. **(C)** Normalized R/S ratios calculated for CDRs and FWRs within each subset. Expanded B-cells were collapsed to a single representative sequence per clonal family. Subpanel labels report mean ± standard error of collapsed R/S ratios.

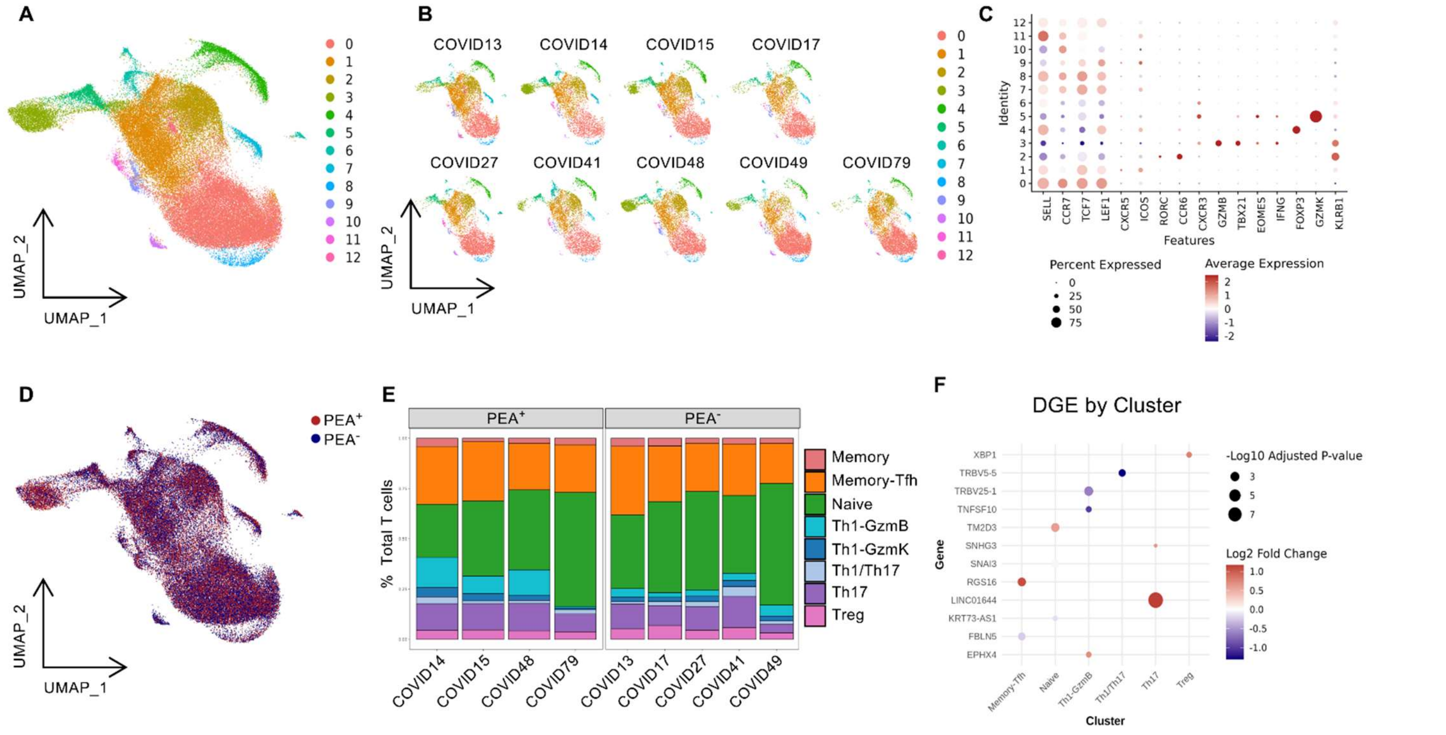

Supplemental Figure 4. T-cell UMAP verification and validation. UMAP verification of T-cell populations and clustering (n = 67,303). **(A)** UMAP showing original T-cell clusters 0–12 across all T-cells. **(B)** UMAP split by individual patient. **(C)** Expression of marker genes used for cluster verification across the 13 original clusters. **(D)** Integration validation by grouping T-cells according to PEA status. **(E)** Distribution of T-cell populations percentages per patient, grouped by PEA status. **(F)** Dotplot of significantly upregulated genes from DESeq2 pseudobulk-corrected differential expression analysis in T-cell clusters comparing PEA^{+} and PEA^{-} patients. Significance was determined using an adjusted p-value cutoff of 0.05.

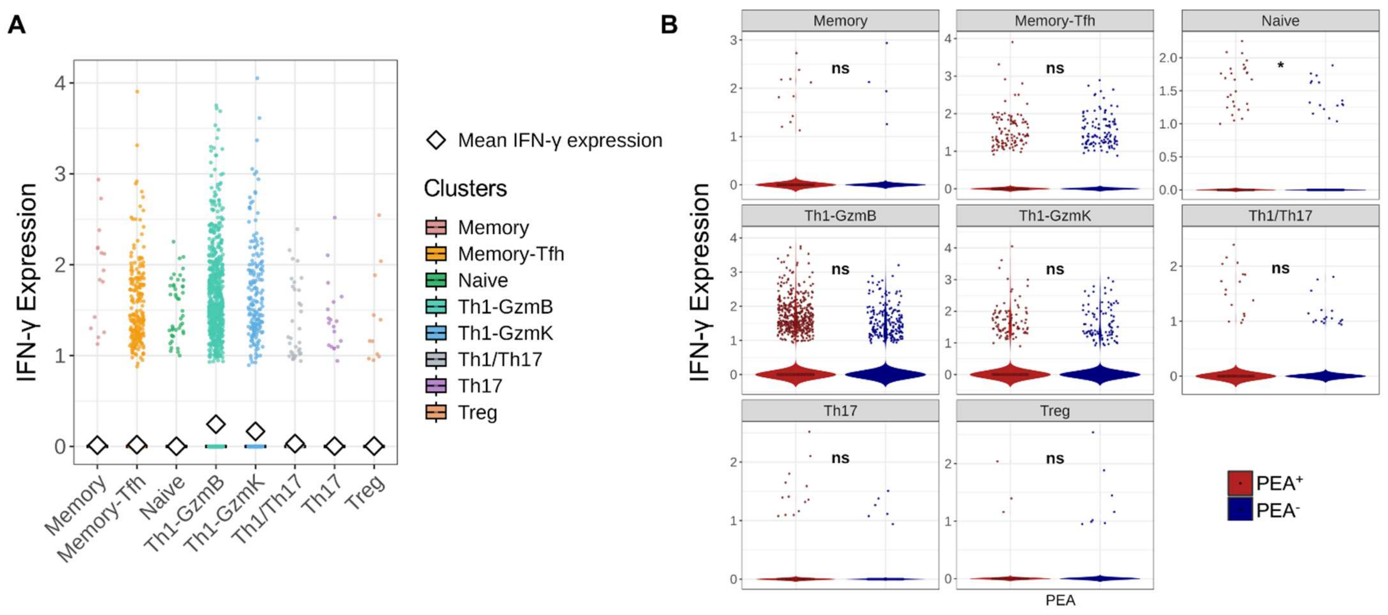

Supplemental Figure 5. IFN-γ expression across T-cell clusters. (A) IFN-γ expression across all T-cell subsets in all patient groups. Colors indicate cluster and white diamonds indicate mean expression within each subset. **(B)** IFN-γ expression across all T-cell subsets and split by PEA status. Statistics were evaluated using Wilcoxon rank-sum test; *p < 0.05, ns = no significance.

Supplemental Tables

Supplemental Table 1. Patient clinical parameters and demographics.

Parameter	PEA^+(n=6)	PEA^-(n=5)	P value
Age, mean(range), y	61.3(45 to 87)	47.8(25 to 73)	0.31
Sex, No.			
Male	5	2	0.16
Female	1	3	
Admission to ICU, No.	1	1	0.82
Deceased, No.	1	1	1
SOFA Score, mean(range)	2.7(1 to 4)	3.2(1 to 6)	0.63
White blood cell count, mean(range), 10^9/L	7.2(5.6 to 8.7)	8.1(4.3 to 11.5)	0.55
Platelet count, mean(range), 10^9/L	256.5(188 to 378)	195.8(153 to 297)	0.15
D-dimer, mean(range), mg/L FEU	1.27(0.34 to 3.6)	6.89(0.24 to 31.16)	0.41
Fibrinogen, mean(range), mg/dL	693.8(506 to 860)	471.8(128 to 660)	0.09
SpO_2 (O_2 saturation), mean(range), %	91.7(88 to 95)	95.8(93 to 97)	0.008
PaO_2, mean(range), mmHg	64.3(54 to 80)	85.6(68 to 110)	0.02
Ferritin, mean(range), ng/mL	619.0(138 to 1122)	377.2(166 to 639)	0.20
C-reactive protein, mean(range), mg/dL	17.0(3.1 to 34.3)	4.48(1.6 to 7.6)	0.04
Alkaline phosphatase, mean(range), IU/L	76.7(65 to 95)	130.0(40 to 292)	0.30
Lactate dehydrogenase, mean(range), U/L	385.5(283 to 512)	357.4(239 to 448)	0.62
Bilirubin, mean(range), mg/dL	0.52(0.2 to 1.5)	3.76(0.2 to 17.2)	0.39
PF4/H IgG, mean(range), OD450	1.38(0.79 to 2.69)	1.47(0.55 to 2.84)	0.86
P-selection expression, mean(range), %	59.69(54.31 to 66.07)	-0.70(-6.08 to 1.88)	7.00×10^{-10}

Supplemental Table 2. Information on B-cells in the scRNA-seq and scV(D)J-seq from each patient.

Patient ID	PEA Status	Total # of Cells in UMAP	# of Cells from Clonal Families with ≥ 7 Cells	# of Cells from Clonal Families with ≥ 3 Cells
COVID13	Neg	4122	130	198
COVID17	Neg	2514	408	714
COVID27	Neg	4512	NA	10
COVID41	Neg	1886	NA	NA
COVID49	Neg	2057	61	123
COVID03	Pos	1884	9	25
COVID06	Pos	3039	NA	3
COVID14	Pos	3640	59	104
COVID15	Pos	2604	14	24
COVID48	Pos	1799	28	40
COVID79	Pos	1448	240	283

Supplemental Table 3. Information on T-cells in the scRNA-seq from each patient.

Patient ID	PEA Status	Total # of Cells in UMAP
COVID13	Neg	8729
COVID17	Neg	5955
COVID27	Neg	7332
COVID41	Neg	6227
COVID49	Neg	8130
COVID14	Pos	6885
COVID15	Pos	8085
COVID48	Pos	8184
COVID79	Pos	7776

Supplemental Table 4. Flow cytometry staining table .

Marker	Fluorophore	Titer/100uL (in uL)	Catalog #	Vendor
CD62L	BV480	5	566174	BD
CD86	PerCP-Cy5.5	5	305419	BioLegend
CD27	PE-Dazzle594	2.5	354921	BioLegend
CD19	PE-Cy7	2.5	302235	BioLegend
CD45	Spark NIR 685	1.25	368552	BioLegend
IgM	BV711	1.25	314539	BioLegend
CXCR3	AF647	1.25	353711	BioLegend
CXCR4	PerCP-e710	1.25	46-9999-41	eBioscience
CD24	PerCP	1.25	311113	BioLegend
CD3	BV510	0.6	563109	BD

CD11c	APC-Fire750	0.6	371509	BioLegend
CD138	APC-R700	0.6	566051	BD
HLA-DR	BV650	0.6	307649	BioLegend
CD95	BV785	0.6	305645	BioLegend
CD8	BUV615	0.6	612994	BD
CD4	BUV563	0.6	51-9016605	BD
CD14-DUMP	BUV395	0.6	563562	BD
CD23	APC	0.3	338514	BioLegend
IgD	BV605	0.3	348231	BioLegend
CD21	PE-Dazzle594	0.3	354921	BioLegend
CD38	BB515	0.3	564499	BD
CXCR5	PE	0.3	356903	BioLegend
IgG	BV421	0.15	410703	BioLegend

Supplemental References:

1. Aran D, Looney AP, Liu L, et al. Reference-based analysis of lung single-cell sequencing reveals a transitional profibrotic macrophage. *Nature Immunology*. 2019;20(2):163-172.
